# Effect of Aqueous Extract of *Crocus sativus* L. on Morphine-Induced Memory Impairment

**DOI:** 10.1155/2012/494367

**Published:** 2012-10-10

**Authors:** Sayede Maryam Naghibi, Mahmoud Hosseini, Fatemeh Khani, Motahare Rahimi, Farzaneh Vafaee, Hassan Rakhshandeh, Azita Aghaie

**Affiliations:** ^1^Neuroscience Research Center and Department of Physiology, School of Medicine, Mashhad University of Medical Sciences, Mashhad, Iran; ^2^Pharmacological Research Center of Medicinal Plants and Department of Pharmacology, School of Medicine, Mashhad University of Medical Sciences, Mashhad 9177948564, Iran

## Abstract

In the present study, the effect of aqueous extracts of saffron on morphine-induced memory impairment was investigated. On the training trial, the mice received an electric shock when the animals were entered into the dark compartment. Twenty-four and forty-eight hours later, the time latency for entering the dark compartment was recorded and defined as the retention trial. The mice were divided into (1) control, (2) morphine which received morphine before the training in the passive avoidance test, (3–5) three groups treated by 50, 150 and 450 mg/kg of saffron extract before the training trial, and (6 and 7) the two other groups received 150 and 450 mg/kg of saffron extract before the retention trial. The time latency in morphine-treated group was lower than control (*P* < 0.01). Treatment of the animals by 150 and 450 mg/kg of saffron extract before the training trial increased the time latency at 24 and 48 hours after the training trial (*P *< 0.05 and *P *< 0.01). Administration of both 150 and 450 mg/kg doses of the extract before retention trials also increased the time latency (*P *< 0.01). The results revealed that the saffron extract attenuated morphine-induced memory impairment.

## 1. Introduction

*Crocus sativus* L. is a plant with green and hairy leaves and funnel-shaped reddish-purple flowers, which is cultivated in some countries including China, Spain, Italy, Greece, and especially Iran. It is commonly known as saffron or “Zaaferan” in Iran and is added to food for its color and taste [1, 2]. The part used for medication is the central part of the flower or the female sexual organ which is also called stigma or style. The main active constituents of this plant are picrocrocin and its derivatives include safranal, flavonoid derivatives, and crocin [3]. Safranal is the main aromatic component of saffron which comprises about 60% of the volatile ingredients in saffron [4]. *Crocus sativus* is used in folk medicine as an antispasmodic, eupeptic, anticatarrhal, carminative, diaphoretic, expectorant, stimulant, stomachic, aphrodisiac, emmenagogue gingival, and sedative [3].

It has been reported that extracts of *Crocus sativus* prevent from scopolamine and ethanol-induced memory impairment in Morris water maze and passive avoidance tests. It also protects against ethanol-induced inhibition of hippocampal long-term potentiation (LTP) [5, 6]. In addition, it has been reported that crocin counteracts the ethanol inhibition of NMDA receptor-mediated responses in rat hippocampal neurons [7]. It has been also shown that saffron attenuates cerebral ischemia [8] and reduces the extracellular hippocampal levels of glutamate and aspartate [9]. Saffron extracts or its active constituents have other activities on the central nervous system including antidepressant [10, 11], anticonvulsant [12, 13], and anxiolytic and hypnotic [14]. Some actions of saffron on central nervous system have been attributed to its effects on opioid system [12]. It has been also demonstrated that aqueous and ethanolic extracts of *Crocus sativus* stigma and its constituent crocin can suppress morphine withdrawal syndrome [1]. 

Learning and memory in laboratory animals are known to be affected by opioids and their antagonists [15]. For example, pretraining administration of morphine impairs memory retrieval in passive avoidance tests which will be restored by pretest administration of the same dose of morphine [16]. Hippocampus is one of the areas involved in learning and memory in which both opioid peptides and opioid receptors are expressed [17]. Endogenous opioid peptides consider important neuromodulators in the brain, which are rich in the hippocampus and cerebral cortex [18]. Using different animal models, it was shown that repeated administering morphine can impair memory and learning processes [17, 19, 20]. 

With regard to the effects of saffron on learning and memory and its interactions with opioid system, the aim of the present study was to evaluate the effect of aqueous extract of *Crocus sativus* L. on morphine-induced memory impairment in mice using the passive avoidance test.

## 2. Materials and Methods

### 2.1. Preparing the Plant Extract

In this study, saffron was kindly provided by Novin Zaafran Company, Mashhad, Iran. The powder (100 g) of saffron was extracted with distilled water in a Soxhlet apparatus for 72 h. The resulting extract was concentrated under reduced pressure and kept at −20°C until being used (yielded 33.2%). The extract was dissolved in saline and was then applied [21–23].

### 2.2. Animals and the Experimental Protocol

Seventy-two male mice (30 ± 5 g, 10 weeks old) were kept at 22 ± 2°C and 12 h light/dark cycle (light on at 7:00 AM). All behavioral experiments were carried out between 10 AM and 2 PM The experiments were conducted in accordance with the Guide for the Care and Use of Laboratory Animals and the study was approved by Mashhad University of Medical Sciences. In the present study, the effects of the extract were examined in 2 experiments. In Experiment 1, the extract was injected before training phase (pretraining effect), while in Experiment 2 the extract was administered 24 and 48 hours after training phase (before the test phase, pretest effect).

**Experiment 1**

In this experiment the pretraining effect of the extract was examined. The animal groups were as follows. (1) Control group (*n* = 8): the animals in this group received saline instead of both the saffron extract and morphine. (2) Morphine group (*n* = 8): the animals were treated by saline instead of saffron extract, but morphine was injected to them (5 mg/kg, s.c.) 30 min before the training phase. (3–5) Pretraining treated groups (*n* = 8 in each group) (pretrain 50, pretrain 150, and pretrain 450): the animals in these groups were daily treated by 50, 150, and 450 mg/kg of saffron extract (i.p.), respectively, for 3 days before the training phase.

**Experiment 2**

In this experiment the pretest effect of the extract was examined. The animal groups were as follows. (1) Control group (*n* = 8): the animals in this group received saline instead of both saffron extract and morphine. (2) Morphine group (*n* = 8): the animals were treated by saline instead of saffron extract before the retention phase, but morphine was injected to them (5 mg/kg, s.c.) 30 min before the training phase. (3–4) Pretest groups (*n* = 8 in each group) (pretest 150 and pretest 450): the animals in these groups received 150 and 450 mg/kg of saffron extract (i.p.), respectively, before the test phase (24 and 48 hours after training phase).

### 2.3. Behavioral Procedures

The animals were handled for 1week before starting the experiments. A passive avoidance learning test based on negative reinforcement was used to examine the memory. The apparatus consisted of a light and a dark compartment with a grid floor adjoining each other through a small gate. The animals were accustomed to the behavioral apparatus during two consecutivedays (5 min in each day) before the training session. On the third day, the animals were placed in the light compartment, and the time latency for entering the dark compartment was recorded. In the training phase, the mice were placed in the light compartment facing away from the dark compartment. When the animals were entered completely into the dark compartment, they received an electric shock (1 MA, 2 s duration). The mice were then returned to their home cage. Twenty-four and forty-eight hours later (the retention phase or test phase), the animals were placed in the light compartment, and the time latency for entering the dark compartment as well as the time spent by the animals in the dark and light compartments was recorded and defined as the retention trial [24, 25]. 

### 2.4. Statistical Analysis

 The data were expressed as mean ± SEM. The statistical analysis was done by one-way ANOVA followed by a tukey post hoc comparison test. The criterion for statistical significance was considered (*P* < 0.05).

## 3. Results

**Experiment 1**

**Figure 1:**
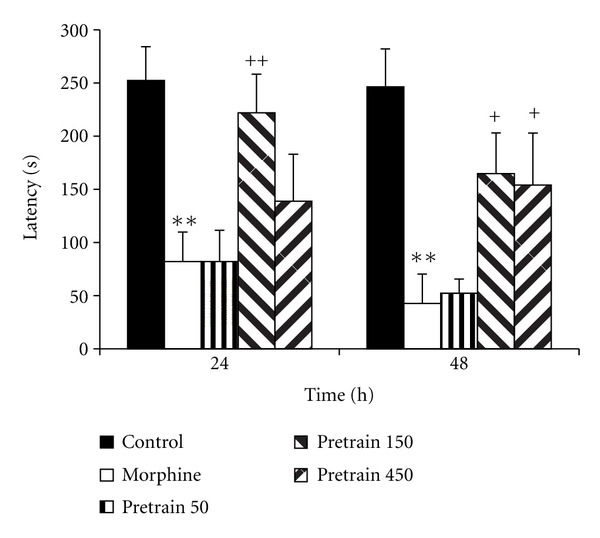
The effects of pretraining injection of saffron extract on time latency for entering the dark compartment 24 and 48 h after receiving the shock in the experimental groups. The animals of control group received saline instead of both saffron extract and morphine. The animals of morphine group were treated by saline instead of SE but received morphine (5 mg/kg, s.c.) 30 min before the training phase. Pretrain 50, pretrain 150, and pretrain 450 groups were treated by 50, 150, and 450 mg/kg of SE (i.p.), respectively, for 3 days before the training phase. The data were presented as mean ± SEM of the time latency (8 animals in each group). **P < 0.01 compared with the control group; ^+^ *P* < 0.05 and ^++^ *P* < 0.01 compared with the morphine group.

**Figure 2:**
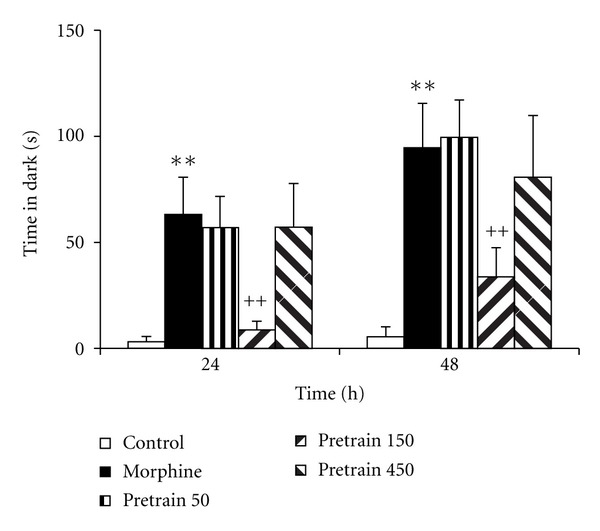
The effects of pretraining injection of saffron extract on the time spent in the dark compartment 24 and 48 h after receiving the shock. The animals of control group received saline instead of both saffron extract and morphine. The animals of morphine group were treated by saline instead of SE but received morphine (5 mg/kg, s.c.) 30 min before the training phase. Pretrain 50, Pretrain 150, and pretrain 450 groups were treated by 50, 150, and 450 mg/kg of saffron extract (i.p.), respectively, for 3 days before the training phase. The data were presented as mean ± SEM of the total time spent in the dark compartment (8 animals in each group). **P < 0.01 compared with the control group; ^++^ *P* < 0.01 compared with the morphine group.

**Figure 3:**
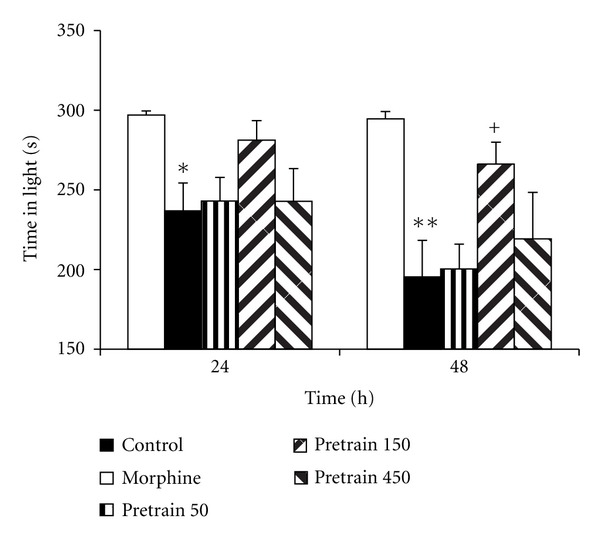
The effects of pretraining injection of saffron extract on the total time spent in the light compartment 24 and 48 h after receiving the shock between the groups. The animals of control group received saline instead of both saffron extract and morphine. The animals of morphine group were treated by saline instead of SE but received morphine (5 mg/kg, s.c.) 30 min before the training phase. Pretrain 50, pretrain 150, and pretrain 450 groups were treated by 50, 150, and 450 mg/kg of SE (i.p.), respectively, for 3 days before the training phase. The data were presented as mean ± SEM of the total time spent in the light compartment (8 animals in each group). *P < 0.05 and **P < 0.01 compared to control; ^+^ *P* < 0.05 compared to morphine group.

In the morphine group, the time latency for entering the dark compartment was lower than that of the control group (Figure 1, *P* < 0.01). The treatment of the animals by 150 and 450 mg/kg of saffron extract significantly increased the time latency for entering the dark compartment Twenty-four and forty-eight h after receiving a shock (Figure 1, *P* < 0.05 and *P* < 0.01). Administration of 50 mg/kg of saffron extract was not effective for changing the time latency for entering the dark compartment. The results also showed that the total time spent in the dark compartment by the animals of the morphine group was higher than that of the saline group (Figure 2, *P* < 0.01). Twenty-four and forty-eight h after receiving the shock, the total time spent in the dark compartment by the animals of the pretrain 150 group was lower than that of the morphine group (Figure 2, both *P* < 0.01). The results also indicated that the total time spent in the light compartment by the animals of the morphine group was lower than that of the saline group at 24 and 48 h after receiving the shock (Figure 3, *P* < 0.05 and *P* < 0.01). In the pretrain 150 group, the total time spent in the light compartment was higher than that of the morphine group at 48 h after receiving the shock (Figure 3, *P* < 0.05).

**Experiment 2**

**Figure 4:**
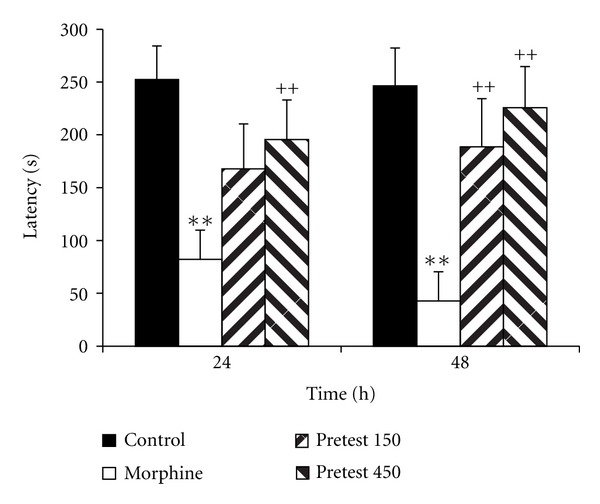
The effects of pretest injection of saffron extract on time latency for entering the dark compartment. The animals of the control group received saline instead of both saffron extract and morphine. The animals of morphine group were treated by saline instead of SE but received morphine (5 mg/kg, s.c.) 30 min before the training phase. Pretest 150 and pretest 450 groups were treated by 150 and 450 mg/kg of SE (i.p.), respectively, before the recall phase. The data were presented as mean ± SEM of the time latency (8 animals in each group). **P < 0.01 compared with the control group and ^++^ *P* < 0.01 compared with the morphine group.

**Figure 5:**
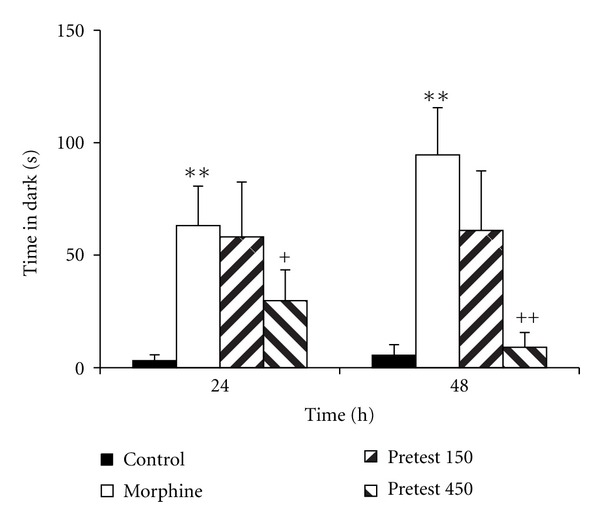
Comparison of the total time spent in the dark compartment in the experimental groups. The animals of the control group received saline instead of both saffron extract and morphine. The animals of morphine group were treated by saline instead of SE but received morphine (5 mg/kg, s.c.) 30 min before the training phase. Pretest 150 and pretest 450 groups were treated by 150 and 450 mg/kg of SE (i.p.), respectively, before the recall phase. The data were presented as mean ± SEM of the total time spent in the dark compartment (8 animals in each group).  **P < 0.01 compared with the control; ^+^ *P* < 0.05 and ^++^ *P* < 0.01 compared with the morphine group.

**Figure 6:**
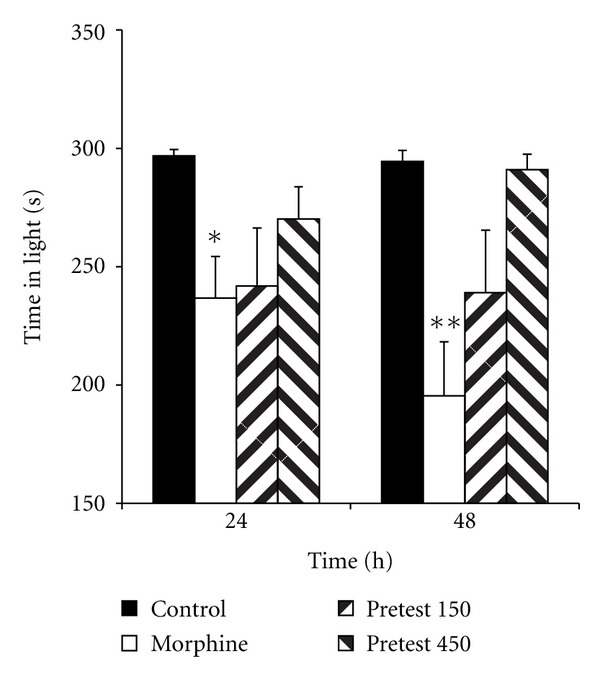
Comparison of the total time spent in the light compartment 24 and 48 h after receiving the shock between the groups. The animals of the control group received saline instead of both saffron extract and morphine. The animals of morphine group were treated by saline instead of SE but received morphine (5 mg/kg, s.c.) 30 min before the training phase. Pretest 150 and pretest 450 groups were treated by 150 and 450 mg/kg of SE (i.p.), respectively, before the recall phase. The data were presented as mean ± SEM of the total time spent in the light compartment (8 animals in each group).

Treatment of the animals by 150 and 450 mg/kg of saffron before retention phases (24 and 48 h after the shock) increased the time latency for entering the dark compartment (Figure 4, *P* < 0.01). The total time spent in the dark compartment by the animals treated by 450 mg/kg of saffron extract was lower than that of the morphine group (Figure 5, *P* < 0.05 and *P* < 0.01). However, there was no significant difference in the total time spent in the light compartment between these groups (Figure 6).

## 4. Discussion

The results of the present study showed that saffron extract attenuated memory impairment induced by morphine. The results were in agreement with the results of previous studies showing the beneficial effects of saffron on memory [5, 26, 27]. Haghighizad et al. also suggested the protective effect of the saffron extract against morphine-induced inhibition of spatial learning and memory in rats. The used doses were lower than that in the present study [28]. It has been recently found that the alcoholic extract of the pistils of *Crocus sativus *L. affects learning and memory in mice [27]. In another study, oral administration of 125–500 mg/kg of *Crocus sativus* extract alone had no effect on the learning behavior of mice in passive avoidance test but significantly improved ethanol-induced impairment of memory acquisition [27]. It has been also shown that treatment of animals by 50–200 mg/kg crocin alone had no effect but significantly improved ethanol-induced impairment of memory acquisition in mice [5]. Intracerebroventricular administration of crocin significantly prevented from ethanol-induced inhibition of hippocampal LTP in anaesthetized rats *in vivo* [26, 29]. The results of the present study also showed that *Crocus sativus* extract inhibited of the deleterious effect morphine on memory.

There is accumulating evidence that opiates modulate synaptic transmission and plasticity in the brain. Opiates have been shown to alter glutamatergic transmission [30], neurogenesis [31], dendritic stability [32], and long-term potentiation [33–35]. The passive avoidance paradigm used in the present study depends upon both the amygdala and hippocampal systems [36–38]. It has been also shown that both the amygdala and hippocampus are involved in the effects of opioids on memory [39–41]. The exact mechanism(s) of morphine induced impairment of memory formation have not been completely elucidated [42]. The role of NMDA receptors in morphine state-dependent learning has been suggested [43, 44]. These receptors are also involved in post-training memory processing by the amygdala and hippocampus [45]. Furthermore, NMDA receptors may have a role in the effect of saffron or its constituents on memory [46, 47]. Moreover, the analgesic effect of saffron is attenuated by NMDA receptor antagonists [48]. It has been suggested that opioid-induced impairment of memory formation may be accompanied by a decreased activity level in nitric oxide/cyclic guanosine monophosphate (NO/cGMP) signaling pathway [16]. The interaction of saffron with nitric oxide has been also reported [49]. It has been shown that morphine-induced memory recall might be influenced by the central cholinergic activity [50]. Beneficial effects of saffron on memory in inhibited cholinergic system animal models [2, 51] may be another explanation for the effect of the extract on memory in the present study. The sedative- effects of as well as the protective effects in pentylenetetrazole (PTZ-) induced seizure in mice and rats may imply that saffron affects the GABAergic system [12, 27, 52] in which the latter has a role in memory impairment by the opioids [53, 54]. It is believed that saffron extract, crocetin or crocin, could be useful in the treatment of brain neurodegenerative disorders because of its powerful antioxidant activities [55]. Considering the possible role of oxidative damage in the deleterious effect of morphine [56], the antioxidant activity of the extract can be considered as another explanation for the beneficial effects of saffron on morphine-induced memory impairments [56, 57]. A functional interaction between dopamine and opioid system in memory storage processes has been suggested [58]. This mechanism may also have a role in the effect of saffron on memory impairment by opioids [59]. There are also other pieces of evidence which confirm the interaction of the opioid system with saffron or its constituents. It has been indicated that crocin produces a dose-dependent antinociceptive effect and also increases morphine-induced antinociception [60]. The analgesic effects of saffron ethanolic extract have been attributed to its effect on the opioid system [48]. Hosseinzadeh and Jahanian. also showed that the aqueous extracts in doses 80–320 mg/kg and ethanolic ones in doses 400–800 mg/kg attenuated morphine withdrawal signs induced by naloxone in mice [1]. It was also shown that injection of 100 mg/kg of *Crocus sativus* extract inhibited the acquisition and expression of morphine-induced conditioned place preference [61, 62]. In the present study, 50–450 mg/kg of the saffron extract inhibited morphine-induced memory impairment; however, it seems that the medium dose was more effective. The results of the present study added evidence for the effects of saffron on the brain opioid system.

In the present study, the chemical compound(s) for the beneficial effects of saffron was not indentified. The presence of crocin as a water-soluble carotenoid, as well as the monoterpene aldehyde and its glucosides including safranal and picrocrocin, and flavonoids including quercetin and kaempferol in saffron, has been well documented [63]. The beneficial effects of saffron on memory have been repeatedly attributed to crocin [7, 51, 55, 63]. Therefore, the results of present study may at least in part be due to this component. However, the memory enhancing effect as well as the protective effects of safranal on PTZ-induced seizure model may also be a good evidence for the interaction of safranal with GABAergic system [6, 12, 13]. It was also shown that safranal affects the extracellular levels of glutamate and aspartate in hippocampal tissues of kainic acid-treated rats [9]. On the other hand, the interaction of safranal with opioid system has been suggested [1, 62]. For example it has been shown that safranal affects morphine-induced conditioned place preference [62]. The injection of safranal to morphine-dependent animals also acted as an opioid antagonist and induced-morphine withdrawal like behaviors including jumping, seizure, diarrhea, ptosis, irritability, and wet-dog shake [1]. Therefore, it seems that this constituent may also have a role in the results of present study. The precise compound(s) and mechanism(s) responsible for the efficacy of saffron extract on memory impairments elicited by morphine still remain as an important issue and need to be clarified by further studies.

## 5. Conclusion

The results of the present study showed that the aqueous extract of *Crocus sativus* prevented from morphine induced memory deficits in mice. Further studies are needed to confirm this protective effect of *Crocus sativus*.
